# Simultaneous expression of MMB-FOXM1 complex components enables efficient bypass of senescence

**DOI:** 10.1038/s41598-021-01012-z

**Published:** 2021-11-02

**Authors:** Ruchi Kumari, Holger Hummerich, Xu Shen, Martin Fischer, Larisa Litovchick, Sibylle Mittnacht, James A. DeCaprio, Parmjit S. Jat

**Affiliations:** 1 MRC Prion Unit at UCL, UCL Institute of Prion Diseases, 33 Cleveland Street, London, W1W 7FF UK; 2 The UCL Cancer Institute, Paul O’Gorman Building, 72 Huntley Street, London, WC1E 6DD UK; 3 Computational Biology Group, Leibniz Institute on Aging – Fritz Lipmann Institute (FLI), Beutenbergstraße 11, 07745 Jena, Germany; 4 Division of Hematology, Oncology and Palliative Care, Department of Internal Medicine, Massey Cancer Centre, Virginia Commonwealth University, Richmond, VA 23298 USA; 5 Department of Medical Oncology, Dana-Farber Cancer Institute, 450 Brookline Avenue, Boston, MA 02215 USA; 6 Department of Medicine, Brigham and Women’s Hospital and Harvard Medical School, 450 Brookline Avenue, Boston, MA 02115 USA

**Keywords:** Cancer, Cell biology, Molecular biology, Oncology

## Abstract

Cellular senescence is a stable cell cycle arrest that normal cells undergo after a finite number of divisions, in response to a variety of intrinsic and extrinsic stimuli. Although senescence is largely established and maintained by the p53/p21^WAF1/CIP1^ and pRB/p16^INK4A^ tumour suppressor pathways, the downstream targets responsible for the stability of the growth arrest are not known. We have employed a stable senescence bypass assay in conditionally immortalised human breast fibroblasts (CL3^EcoR^) to investigate the role of the DREAM complex and its associated components in senescence. DREAM is a multi-subunit complex comprised of the MuvB core, containing LIN9, LIN37, LIN52, LIN54, and RBBP4, that when bound to p130, an RB1 like protein, and E2F4 inhibits cell cycle-dependent gene expression thereby arresting cell division. Phosphorylation of LIN52 at Serine 28 is required for DREAM assembly. Re-entry into the cell cycle upon phosphorylation of p130 leads to disruption of the DREAM complex and the MuvB core, associating initially to B-MYB and later to FOXM1 to form MMB and MMB-FOXM1 complexes respectively. Here we report that simultaneous expression of MMB-FOXM1 complex components efficiently bypasses senescence with LIN52, B-MYB, and FOXM1 as the crucial components. Moreover, bypass of senescence requires non-phosphorylated LIN52 that disrupts the DREAM complex, thereby indicating a central role for assembly of the DREAM complex in senescence.

## Introduction

Cellular senescence is a stable cell cycle arrest that normal cells undergo after a finite number of divisions, known as the Hayflick limit^1^. Senescence is triggered in response to a variety of intrinsic and extrinsic stimuli including progressive shortening of telomeres and other changes in telomeric structure, as well as various forms of stress that lead to a persistent DNA damage response such as oncogene activation and oxidative stress. Senescence is largely established and maintained by engaging either one or both p53/p21^WAF1/CIP1^ and pRB/p16^INK4A^ tumour suppressor pathways. Both pathways involve many upstream regulators and downstream effectors along with various interlinked side branches^2–6^.

Previously, we identified several genes including some components of the DREAM complex and its associated factors that are differentially expressed in cellular senescence^7^. DREAM is a multi-subunit complex, formed by the assembly of p130 or p107 (RB family of pocket proteins) together with Dimerization partner 1–2 (DP), E2F4-5, and a Multivulval class B (MuvB) core^8^. The MuvB core comprises LIN9, LIN37, LIN52, LIN54, and RBBP4^9^. During G0/G1, the MuvB core binds to p130/p107 and E2F4/DP to form the DREAM complex, which inhibits cell cycle-dependent gene expression thereby arresting cell division. When cells exit G0/G1, phosphorylation of p130/p107, leads to dissociation from MuvB and E2F^10^, allowing activator E2Fs to upregulate genes required for progression into S phase. Upon entry into the cell cycle, the MuvB core binds to B-MYB to form the MMB activator complex and regulate late S phase genes. In G2 phase, MMB recruits FOXM1; this is followed by proteasomal degradation of B-MYB, whereas active FOXM1 remains bound to MuvB and regulates expression of genes required for the G2/M transition^9,11,12^.

Although a role for the DREAM complex in cellular senescence has not been widely studied, it has been shown to contribute to Ras-induced senescence^13^. Here we have examined the role of the DREAM complex and its associated components in senescence using human breast fibroblasts (CL3^EcoR^) conditionally immortalised using a thermolabile SV40 large T (LT) antigen (U19tsA58) along with the catalytic subunit of human telomerase (hTERT)^14^. These cells are immortal if grown at 33.5 ± 0.5 °C, but undergo a stable growth arrest within 7 days upon inactivation of the thermolabile LT antigen at the non-permissive temperature 39 ± 0.5 °C. At the restrictive temperature, these inducibly senescent cells acquire classical features of senescence including changes in morphology, appearance of senescence-associated β-galactosidase, and expression of genes shared by other senescent cells^7,14,15^. Senescence can be bypassed in these cells by inactivation of the p53/p21^WAF1/CIP1^ and pRB/p16^INK4A^ pathways^7^. Recently it was shown that overexpression of B-MYB or expression of a non-phosphorylated form of LIN52-S28 disrupts DREAM assembly, promotes MMB formation, and upregulates FOXM1^13,16^. Here we show that simultaneous expression of MMB-FOXM1 complex components, allowed cells to bypass senescence efficiently and that the critical components are: non-phosphorylated LIN52, B-MYB and an active FOXM1.

## Results

### Reconstitution of the DREAM associated MMB-FOXM1 complex bypasses senescence

**Figure 1:**
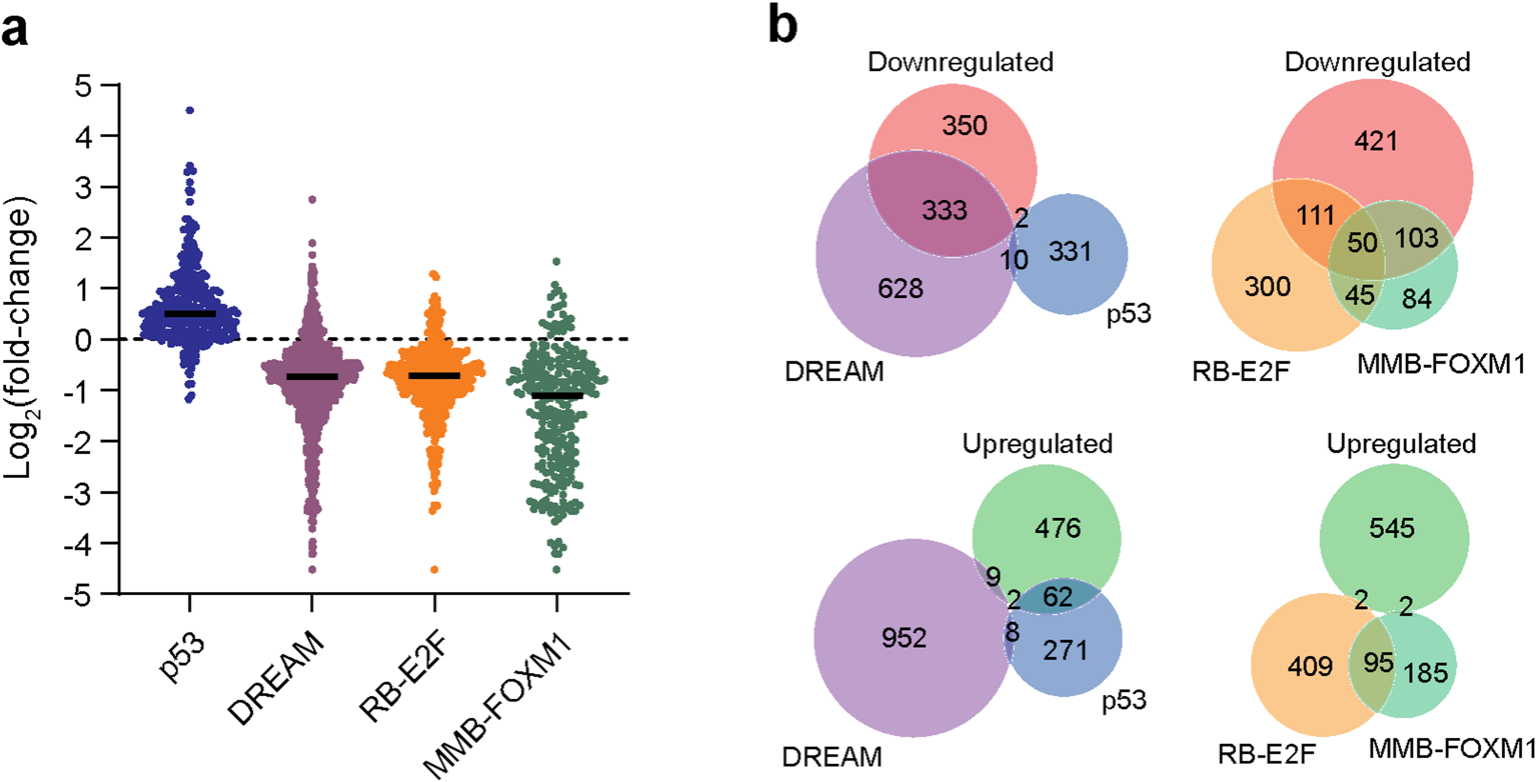
Senescence associated differential gene expression. (**a**) Differential expression of p53^18^, DREAM, RB-E2F, and MMB-FOXM1 target genes^19^ in CL3^EcoR^ cells following senescence-induced growth arrest. Black line indicates the median. (**b**) Venn diagram of overlap between up and downregulated genes, whose expression was reversed upon senescence bypass, and p53, DREAM, RB-E2F, and MMB-FOXM1 target genes.

Previously, we used expression profiling to identify genes differentially expressed upon cellular senescence in the human CL3^EcoR^ fibroblasts^7^. We found that senescence-induced growth arrest was associated with increased expression of p53 target genes, whereas expression of target genes of the DREAM, RB-E2F, and MMB-FOXM1 complexes were decreased (Fig. 1a), supporting an important role for the p53/p21^WAF1/CIP1^- DREAM/RB-E2F and p16^INK4A^/RB signalling pathways in establishing the senescence phenotype. We identified 549 up-regulated and 685 down-regulated genes whose expression was reversed when senescence was bypassed by inactivation of the p53/p21^WAF1/CIP1^ and pRB/p16^INK4A^ pathways^7^. When these genes were overlapped with published lists of p53, DREAM, RB-E2F, and MMB-FOXM1 target genes^17^, we found that DREAM (49%), RB-E2F (16%), and MMB-FOXM1 (22%) targets indeed accounted for a large proportion (87%) of the downregulated genes (Fig. 1b and Supplementary Table 1). Direct p53 target genes, however, accounted for only a small fraction (12%) of the highly upregulated genes, indicating an involvement of other transcription factors or indirect pathways. These findings further suggested that re-expression of cell cycle genes regulated by the DREAM, RB-E2F, and MMB-FOXM1 complexes may be key for bypassing senescence.

**Figure 2:**
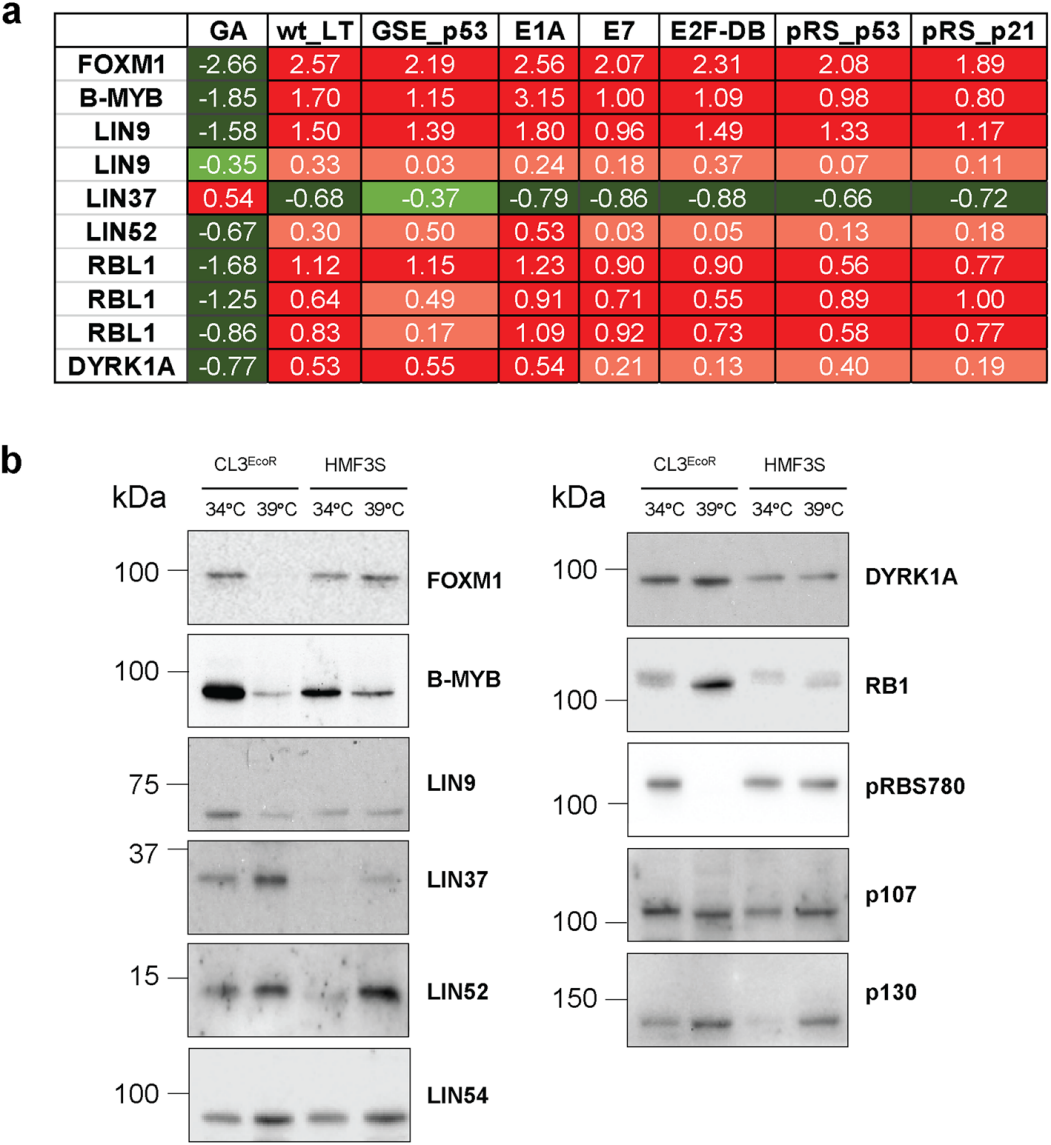
Senescence growth arrest in CL3^EcoR^ cells. (**a**) Senescence specific changes in expression at the RNA level as determined by expression profiling are shown. GA indicates Log2 fold changes in RNA expression upon senescence growth arrest. Positive numbers for GA indicate up-regulation and are shaded in red, (log2 fold change less than 0.5 are shaded in light red). Negative numbers indicating down-regulation are shaded in green (log2 fold change less than − 0.5 are shaded in light green). Fold changes in expression of LIN9 and RBL1 are shown for 2 and 3 independent features whereas all the others are for a single feature only. Also shown is the effect on expression when senescence was bypassed by inactivation of the pRB/p16^INK4A^ and p53/p21^WAF1/CIP1^ pathways^7^. The pRB/p16^INK4A^ pathway was inactivated using SV40 wild type LT (wt_LT), Adenovirus 5 E1A 12S (E1A), Human Papilloma Virus (HPV) 16 E7 (E7) or a dominant negative E2F-DB protein^7,21^. The p53/p21^WAF1/CIP1^ pathway was inactivated using SV40 wild type LT, a p53GSE element that inactivates p53 (GSE-p53)^22^ or short-hair pin RNAs targetting p53 (pRS_p53)^23^ or p21^WAF1/CIP1^ (pRS_p21)^7^. The changes in expression were reversed upon senescence bypass. (**b**) Representative immunoblots assessing changes in protein levels of FOXM1, B-MYB, LIN9, LIN37, LIN52, LIN54, DYRK1A, RB1, pRB^S780^, p107, and p130 in CL3^EcoR^ and HMF3S cells. Images of uncropped gels and blots are shown in the Supplementary Figures.

LIN9 and LIN52, two components of the MuvB core, along with B-MYB and FOXM1, were significantly downregulated upon senescence whereas LIN37 was upregulated (Fig. 2a); these changes were reversed when senescence was bypassed by inactivation of the p53/p21^WAF1/CIP1^ and pRB/p16^INK4A^ pathways^7^. LIN proteins associate to form the MuvB core which binds to B-MYB upon destabilization of DREAM to assemble the MMB complex and subsequently to FOXM1, to form the MMB-FOXM1 complex^9,20^. Interestingly p107 (RBL1) and Dual specificity tyrosine phosphorylation-regulated kinase 1A (DYRK1A), a kinase that phosphorylates LIN52 at serine 28, were also significantly downregulated (Fig. 2a). We have previously used a Human Signal Transduction Open Array™ to confirm the expression profiling data by simultaneously analysing 600 genes by real—time qPCR^7^. After filtering to eliminate growth arrest genes whose expression was very low (Ct > 22), nearly 80% of the genes showed concordant changes in expression by real time qPCR or expression profiling although the actual Log_2_Fold Changes obtained by the two methods were different^7^.

To determine if changes in gene expression were also reflected by alterations in the protein level, cell lysates prepared from CL3^EcoR^ cells actively dividing at 34 °C and after shift up to 39 °C for 4 days were examined by western blotting. To rule out changes in expression due to the temperature shift, lysates were simultaneously prepared and analysed from HMF3S cells. These cells were derived by immortalisation of the same batch of human breast fibroblasts with a wild type SV40 U19 LT antigen along with hTERT and do not undergo senescence arrest upon shift up 39 °C^15^. Since senescence is associated with changes in cell size, cell lysates equivalent to equal numbers of cells were compared. The results in Fig. 2b show that expression of FOXM1 and LIN9 were clearly reduced upon senescence arrest in accordance with downregulation at the RNA level. Expression of B-MYB was also reduced, while a reduction was also seen in HMF3S cells upon shift up even though the reduction was much greater in the CL3^EcoR^ cells upon senescence. In accordance with the RNA data, LIN37 was clearly upregulated upon senescence whereas LIN54 expression was slightly increased due to the temperature shift. LIN54 RNA was not significantly altered upon senescence. In contrast to the above, the reduction in RNA levels upon senescence arrest for LIN52 and DYRK1A did not translate into a change in protein levels, with western blots revealing similar if slightly higher levels of protein in lysate from cells displaying senescence compared to proliferating cells. In conclusion, many of the changes in RNA for components of the DREAM complex translate into changes at the protein level.

In accordance with the activation of the pRB/p16^INK4A^ pathway playing a central role in the induction of senescence^7,24,25^, we found that RB1 levels were increased upon senescence in CL3^EcoR^ cells and this was associated with loss of RB1 phosphorylation, indicated by overt loss of reactivity with anti RB1 serine 780 antibody (Fig. 2b). Although expression of the RB1-related p107 was clearly reduced at the RNA level upon senescence (Fig. 2a), the protein level was only slightly decreased and the protein appeared to migrate faster, consistent with loss of phosphorylation leading to its activation as a growth suppressor. The level of the RB1- related p130 increased upon senescence in the CL3^EcoR^ cells, but this was also observed in the HMF3S cells indicating that the change in expression was due to the temperature shift. Together these results indicate that the functional engagement of RB1 family of proteins upon cell senescence is not seen in proliferating cells including temperature shifted HMF3S cells.

We have previously found that inactivation of the p53/p21^WAF1/CIP1^ and pRB/p16^INK4A^ pathways in CL3^EcoR^ cells bypasses senescence. Inactivation of the p53/p21^WAF1/CIP1^ pathway with p53GSE that inactivates p53, or shRNAs that target p53 or p21^WAF1/CIP1^ bypassed senescence very efficiently^7,22,23^, as indicated by densely growing colonies observed after plating < 3000 stably transduced cells. In contrast, bypass of senescence was much less efficient upon inactivation of the pRB/p16^INK4A^ pathway^7^ where growing cells were only obtained after plating 35,000–50,000 stably transduced cells. Since disruption of the DREAM complex by ectopic expression of a LIN52 mutant that cannot be phosphorylated at serine 28 (LIN52-S28A) can suppress Ras-induced senescence^13^, our aim was to examine whether disruption of the DREAM complex would contribute to the bypass of senescence in CL3^EcoR^ cells in a long-term senescence bypass assay^22,24,25^, thereby supporting the idea that re-expression of cell cycle genes regulated by the DREAM, RB-E2F, and MMB-FOXM1 complexes may be key for bypassing senescence.

We first tested the potential of the individual components of the MMB-FOXM1 complex to bypass senescence using full-length, wild-type ORFs for B-MYB, LIN9, LIN37, and LIN54 or constitutively active forms of FOXM1 (ΔNΔKEN) and LIN52 (S28A). FOXM1ΔNΔKEN is constitutively active as it lacks the amino terminal auto-inhibitory domain and the KEN box, required for the proteolytic degradation of FOXM1^26^. Substitution of serine 28 with alanine in LIN52-S28A leads to an inability to bind p130 and assemble the DREAM complex thereby interfering with Ras-induced senescence in hTERT immortalized human BJ fibroblasts^8^. Recently, it has been shown that overexpression of B-MYB disrupts the DREAM complex, dependent upon its ability to interact with MuvB^16^. It is not known if overexpression of constitutively active FOXM1 can disrupt the DREAM complex.

**Figure 3:**
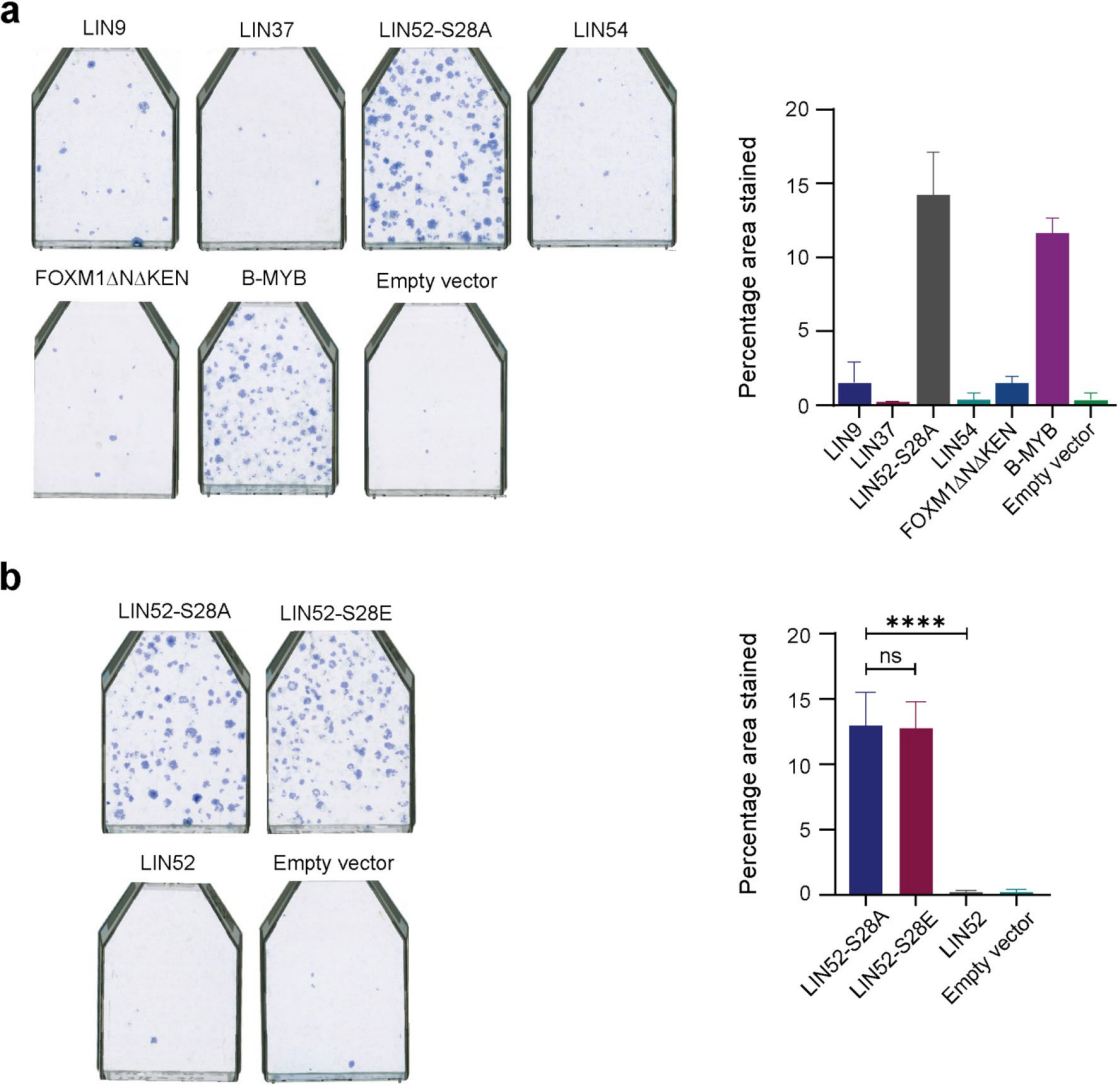
Senescence bypass assays using individual components of the MMB-FOXM1 complex. (**a**) Computational analysis of single representative flasks upon ectopic expression of the indicated component is shown. Blue spots depict the area of the flask computed as blue by a computer algorithm. Quantitative representation of the bypass assay is presented graphically by plotting the calculated percentage area (+ /− SD) covered in blue by Definiens Developer XD software for each of the replicate flasks. Constitutively active LIN52 (LIN52-S28A) and FOXM1 (FOXM1ΔNΔKEN) were utilised. The strongest bypass was exhibited by B-MYB and LIN52-S28A. For a quantitative analysis, the bar chart depicts an overall average (+ /− SD) of the two independent repeat experiments. Empty pLEX-MCS vector was used as the negative control. Statistical analysis was conducted using One-way ANOVA, Tukey’s Multiple Comparison Test (*p < 0.05, **p < 0.01, ***p < 0.001, ****p < 0.0001). (**b**) Comparisons of the senescence bypass potential of wild type LIN52 and mutants thereof. LIN52 was inactive whereas both LIN52 mutants (LIN52-S28A and LIN52-S28E) exhibited senescence bypass activity. For quantitative analysis the bar charts depict an overall average (+ /− SD) of the three independent repeat experiments. Empty pLEX-MCS vector was used as negative control. Statistical analysis was conducted using One-way ANOVA, Tukey’s Multiple Comparison Test (*p < 0.05, **p < 0.01, ***p < 0.001, ****p < 0.0001).

We found that lentiviral (pLEX-MCS) mediated stable transduction of CL3^EcoR^ cells with high titres of either LIN52-S28A and B-MYB produced growing colonies upon plating 35,000 stably transduced cells (Fig. 3a), in marked contrast to a short-hairpin RNA targeting p21^WAF1/CIP1^ which resulted in confluent flasks and a large number of colonies that were too numerous to count as observed previously^7^. Expression of wild type LIN9, LIN37, LIN54 or the constitutively active FOXM1ΔNΔKEN yielded either no colonies or a few colonies similar to the empty vector control. The lack of bypass observed with the FOXM1ΔNΔKEN was rather surprising since we have previously observed that its expression from the retroviral vector, pLPCX, exhibited some bypass potential^7^. However the senescence bypass assays presented here were carried out under more stringent conditions than previously^7^ to minimize background due to leakiness of the U19tsA58 LT antigen.

Phosphorylation of LIN52 at serine 28 by DYRK1A is crucial for regulating the switch between the repressive DREAM complex and the activating MMB/MMB-FOXM1 complexes^13^. Structural analysis has shown that this phosphorylation supports the binding of LIN52 to p130 and p107 allowing assembly of the DREAM complex^27^. To confirm that loss of phosphorylation of Ser-28 in LIN52 was required for bypass of senescence in these CL3^EcoR^ cells, synthetic constructs for LIN52 wild type, LIN52-S28A and LIN52-S28E were prepared, inserted into pLEX-MCS and assayed. The results (Fig. 3b) showed that in contrast to LIN52-S28A, wild type LIN52 was unable to bypass senescence and did not yield any growing colonies, confirming that phosphorylation of serine 28 enabling the assembly of the DREAM complex was required to promote senescence. To further confirm the role of assembly of the DREAM complex in promoting senescence, we replaced Serine 28 in LIN52 with glutamic acid since LIN52-S28E binds p130 very inefficiently^26^. No significant reduction in the number of growing colonies was observed compared to LIN52-S28A (Fig. 3b). Together these results confirmed the importance of the disruption of the repressive DREAM complex for bypassing senescence in these inducibly senescent human cells.

**Figure 4:**
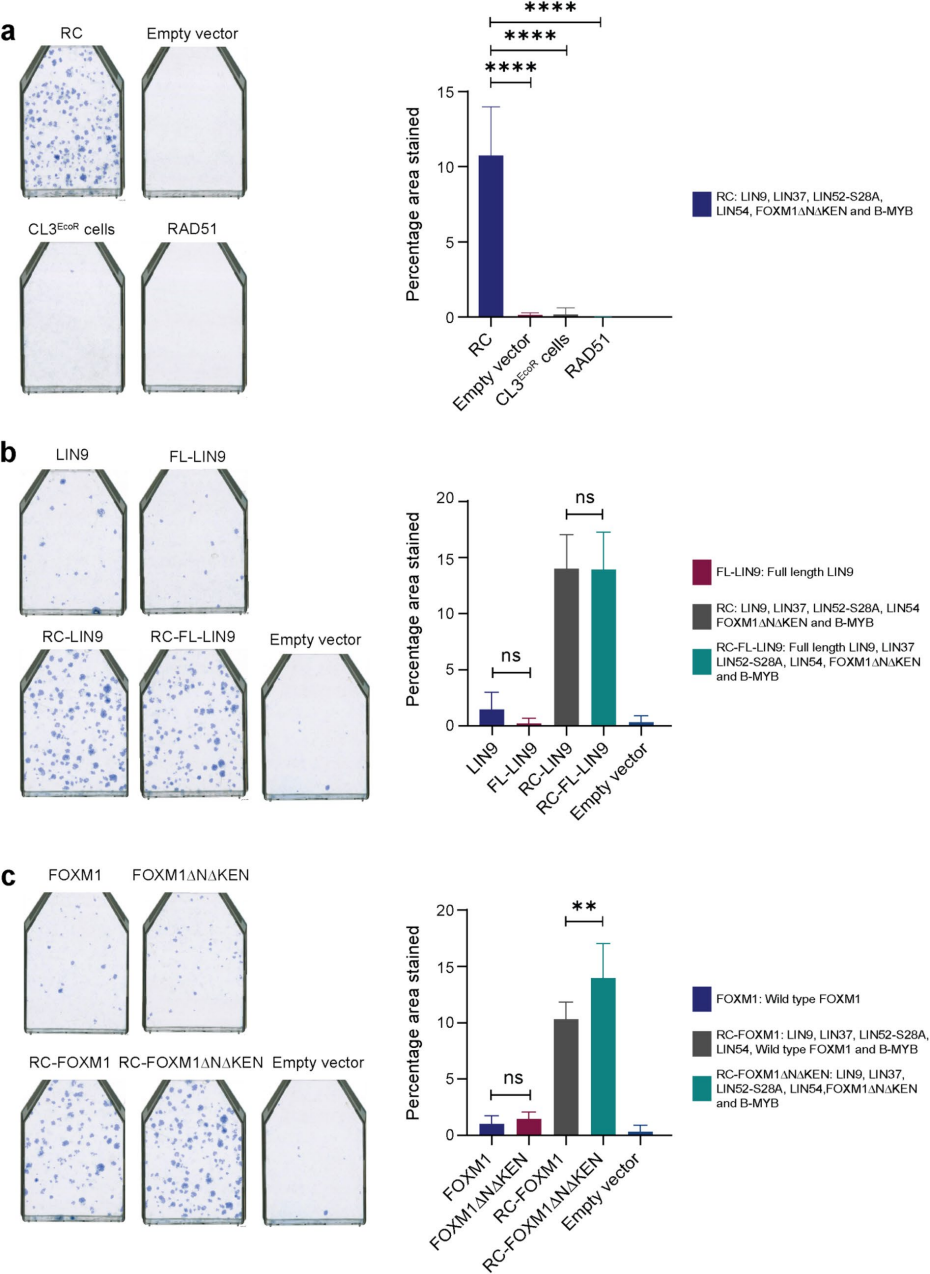
Senescence bypass assays after reconstitution of the MMB-FOXM1 complex. (**a**) Reconstitution with all the components of MMB-FOXM1 complex, indicated as RC, exhibited an efficient senescence bypass. The bar charts depict an overall average (+ /− SD) of three independent repeat experiments. Single representative flasks from the internal repeats are shown. Empty pLEX-MCS vector, CL3^EcoR^ cells and RAD51 were used as negative controls. Statistical analysis was conducted using One-way ANOVA, Tukey’s Multiple Comparison Test (*p < 0.05, **p < 0.01, ***p < 0.001, ****p < 0.0001). (**b**) No significant difference in the senescence bypass potential for full length (FL-LIN9) and the canonical LIN9 (that lacked sixteen amino acids present in FL-LIN9 N-terminus due to a different start codon) was observed when examined individually or as part of the MMB-FOXM1 complex. The bar charts depict an overall average (+ /− SD) of two independent repeat experiments. Empty pLEX-MCS vector was used as a negative control. Statistical analysis was conducted using One-way ANOVA, Tukey’s Multiple Comparison Test (*p < 0.05, **p < 0.01, ***p < 0.001, ****p < 0.0001). (**c**) Reconstitution of the MMB-FOXM1 complex with constitutively active FOXM1 (FOXM1ΔNΔKEN)^26^ exhibited a significantly greater number of colonies than the complex reconstituted with wild type FOXM1 whereas when studied individually, wild type and constitutively active FOXM1 exhibited no significant difference, as very few colonies were obtained. Empty pLEX-MCS vector was used as the negative control. Statistical analysis was conducted using One-way ANOVA, Tukey’s Multiple Comparison Test (*p < 0.05, **p < 0.01, ***p < 0.001, ****p < 0.0001).

Since the number of growing colonies obtained was quite low in comparison to inactivation of the p53/p21^WAF1/CIP1^ pathway, we wanted to determine if transduction of CL3^EcoR^ cells with a mixture of lentiviruses prepared from a pool of components of the MMB-FOXM1 complex would bypass senescence more efficiently compared to single components alone. Lentiviruses were prepared from pools of DNA comprising the different gene constructs at one sixth the amount of DNA used above for the single constructs; used to infect CL3^EcoR^ cells in the senescence bypass assay under very stringent conditions of 39 ± 0.5 °C to minimize background; and identify any potential synergy. Strikingly, a combined transduction of the MMB-FOXM1 complex components, LIN9, LIN37, LIN54, B-MYB, LIN52-S28A, and FOXM1ΔNΔKEN abrogated senescence in CL3^EcoR^ cells very efficiently (Fig. 4a) although not as efficiently as silencing p21^WAF1/CIP1^ which produced confluent flasks within 14 days upon shift up (data not shown), but more efficiently than any of the single components alone. It was also more efficient than inactivation of the pRB/p16^INK4A^ pathway using adenovirus E1A 12S, or human papilloma virus 16 E7, or any other single construct that we have previously tested including LIN52-S28A and B-MYB, other than those that directly inactivate p53 or p21^WAF1/CIP1^^7^. The bypass was highly reproducible under these very stringent assay conditions. A LIN9 (FL-LIN9) construct, corresponding to a predicted LIN9 ORF 16 amino acids longer at the amino terminus than the canonical LIN9^9^ due to a different start codon, was no more efficient than the canonical LIN9 (Fig. 4b). However, expression of the constitutively active FOXM1 was more efficient than wild type FOXM1 for overcoming senescence when combined with MMB components (Fig. 4c).

### LIN52, FOXM1, and B-MYB contribute to bypassing senescence

**Figure 5:**
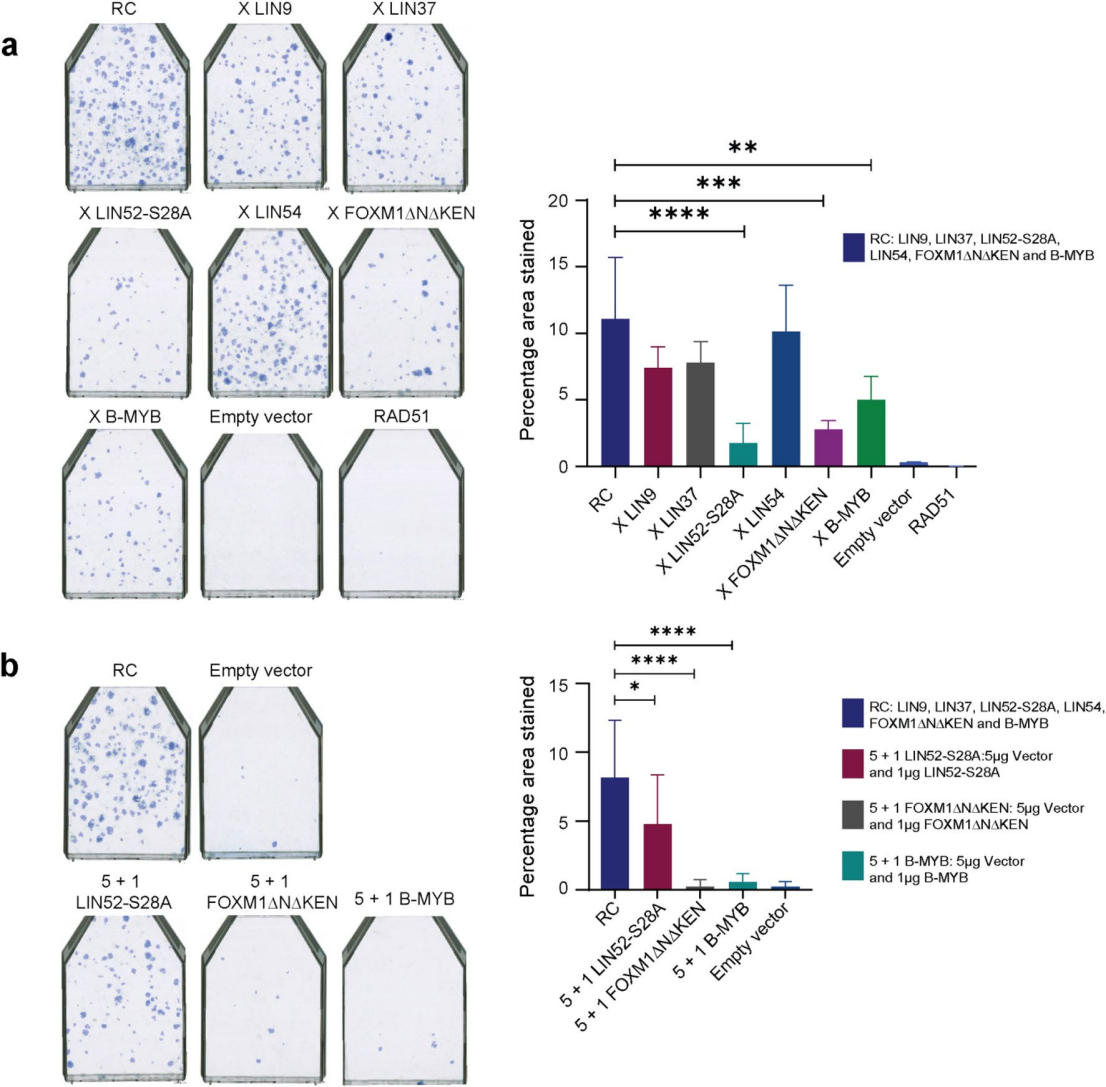
Non-phosphorylated LIN52, FOXM1 and B-MYB are critical for bypassing senescence. (**a**) Bypass assays of the reconstituted MMB-FOXM1 complex lacking one component. The highest numbers of densely growing colonies were observed for the whole reconstituted complex “RC” and “X LIN54” in which LIN54 had been omitted. “X LIN9” and “X LIN37” produced the next highest whereas “X LIN52-S28A”, “X FOXM1ΔNΔKEN” and “X B-MYB” produced the least of which “X LIN52-S28A” lacking LIN52-S28A was the lowest. For qualitative analysis single representative flasks from internal repeats are shown with the component absent from the reconstituted complex as indicated. “X” indicated in front of a component depicts its absence in the reconstituted complex. For quantitative analysis, the bar charts depict an overall average (+ /− SD) of three-independent repeat experiments. Empty pLEX-MCS vector and RAD51 were used as the negative control. Statistical analysis was conducted using One-way ANOVA, Tukey’s Multiple Comparison Test (*p < 0.05, **p < 0.01, ***p < 0.001, ****p < 0.0001). (**b**) LIN52, FOXM1 and B-MYB together synergize to bypass senescence. A significant difference in the bypass potential of the reconstituted MMB-FOXM1 complex was observed with respect to each of the critical components. For quantitative analysis the bar charts depict an overall average (+ /− SD) of three-independent repeat experiments. Empty pLEX-MCS vector was used as a negative control. Statistical analysis was conducted using One-way ANOVA, Tukey’s Multiple Comparison Test (*p < 0.05, **p < 0.01, ***p < 0.001, ****p < 0.0001).

To identify the components of the MMB-FOXM1 complex crucial for the highly efficient bypass, reconstruction experiments were undertaken where one component was left out in turn. The rationale was that if a critical component was omitted, its absence would reduce colony formation. Using this drop-out approach, we observed that absence of LIN52-S28A, FOXM1ΔNΔKEN, or B-MYB led to a significantly reduced bypass of senescence (Fig. 5a). The strongest effect was observed when LIN52-S28A was omitted followed by FOXM1ΔNΔKEN and B-MYB, thereby identifying a key role for these three factors in bypassing senescence. Drop-out of LIN9, LIN37, or LIN54 did not significantly reduce the bypass efficiency in this assay. To identify if there was synergy between the key components, the extent of bypass obtained individually with LIN52-S28A, FOXM1 and B-MYB was compared to the bypass potential of a combination of all the components of the MMB-FOXM1 complex. The highest number of growing colonies was obtained with the complete set of MMB-FOXM1 complex components. There was a clear reduction in the number of colonies obtained when LIN52-S28A, B-MYB and FOXM1ΔNΔKEN were present in the same amount as in the complete MMB-FOXM1 complex (Fig. 5b), suggesting a synergistic effect when MMB-FOXM1 complex components were expressed simultaneously. Essentially no difference in the efficiency of bypass was observed when all the MMB-FOXM1 complex components were compared with a combination of LIN52-S28A, B-MYB and FOXM1ΔNΔKEN (unpublished data).

## Discussion

We have employed a stable senescence bypass assay using conditionally immortalised human breast fibroblasts (CL3^EcoR^) to examine the role of the DREAM complex and its associated components in senescence. DREAM is a multi-subunit complex that inhibits cell cycle-dependent gene expression thereby arresting cell division leading to quiescence. Upon exit from quiescence, the DREAM complex dissociates enabling the MuvB core to bind B-MYB and form the MMB complex for regulating late S phase genes. Later in the cell cycle MMB recruits FOXM1 to regulate expression of genes required for G2 and the G2/M transition^8,10,12^. Here we report that simultaneous expression of MMB-FOXM1 complex components efficiently bypasses senescence and that LIN52, B-MYB and FOXM1 are the crucial components. Moreover, bypass of senescence requires that serine 28 of LIN52 is not phosphorylated, indicating a central role for the DREAM complex in inducing senescence.

FOXM1 and B-MYB are both significantly down-regulated upon senescence in CL3^EcoR^ cells^7^. FOXM1 is also down-regulated upon replicative senescence in primary human fibroblasts^15^. Both B-MYB and FOXM1 play important roles in cell proliferation and cancer^9,28,29^. FOXM1 is a Forkhead transcription factor that regulates G2-specific gene expression, promotes cell proliferation and contributes to tumour progression. B-MYB, a member of the Myb family, is ubiquitously expressed in proliferating cells and a key physiological regulator of cell survival and cell cycle progression^30^. Silencing of B-MYB in primary human foreskin fibroblasts induces senescence^31^, whereas overexpression rescues Ras-induced premature senescence in primary rodent cells^32^ and disrupts the DREAM complex in human cells^16^. B-MYB, upon association with MuvB, binds and regulates transcription of genes containing cell cycle genes homology region (CHR) elements in their promoters^33–35^. As the MMB complex does not contain any E2F proteins, it binds exclusively to the CHR element via LIN54^33,35,36^. The DREAM complex binds to CHR elements in addition to E2F sites and thus has the potential to regulate a larger set of genes than RB and perform regulatory functions distinct from repressive RB/E2F complexes^11,26,34^.

None of the MuvB components, apart from non—Ser-28—phosphorylated LIN52 exhibited a key role towards bypass of senescence (Figs. 3, 5). Phosphorylation of Ser-28 in LIN52 is important since the LxSxExL (aa 26–32) motif of LIN52 binds directly with LxCxE in the pocket domain of p107 and p130 during assembly of the DREAM complex^27^. Phosphorylation of LIN52 at Ser-28 by DYRK1A promotes DREAM assembly, whereas phosphorylation of p107/p130 by CDKs facilitates disassembly to promote cell cycle progression^27^. LIN52 therefore plays a central role in the assembly of repressive DREAM complex. It is also required for MuvB to bind B-MYB to form the MMB activator complex but this is independent of Ser-28 phosphorylation^11,26^. LIN52-S28A disrupts assembly of the DREAM complex thereby interfering with Ras-induced senescence^8^. This is in accordance with our finding that LIN52-S28A promotes bypass of senescence probably by disrupting DREAM and promoting assembly of the MMB complex. It was recently shown that overexpression of B-MYB disrupts the DREAM complex by increasing LIN52 protein levels, dependent upon the MuvB binding domain of B-MYB and the Ser-28 phosphorylation site of LIN52^16^. However it remains to be investigated if overexpression of constitutively active FOXM1 can also disrupt the DREAM complex in a similar manner. This study has shown that simultaneous expression of MMB-FOXM1complex components, particularly dephosphorylated LIN52, B-MYB and active FOXM1, enables an efficient bypass of senescence, by promoting both DREAM disassembly and activation of mitotic gene expression thereby highlighting a central role for the DREAM complex and the importance of the p53/p21-DREAM/RB-E2F and p16/RB pathways in establishing the senescence phenotype.

## Materials and methods

### Cell culture

CL3^EcoR^ and HMF3S cells and derivatives thereof were maintained at 33.5 ± 0.5 °C and temperature shift experiments were performed at 39 ± 0.5 °C. They were developed by our group and have been published previously^7,14,15^. CL3^EcoR^ cells are a single cell clone of HMF3A cells that express the full length murine ecotropic retroviral receptor and closely mirror the parental cells in their temperature dependent growth characteristics^7^. HMF3A cells were developed by conditional immortalisation of freshly isolated mammary fibroblasts using a thermolabile SV40 LT antigen along with hTERT^14^. HMF3S cells were derived by immortalisation of the same batch of human breast fibroblasts as HMF3A cells with a wild type SV40 U19 LT antigen along with hTERT^15^. Breast fibroblasts were prepared from normal breast tissue obtained with written informed consent from patients undergoing cosmetic surgery^14^ in accordance with UCL Institutional guidelines and regulations. All experimental procedures were undertaken in microbiological containment level 2 and level 3 facilities with strict adherence to safety protocols and guidelines. The study was approved by ‘The Joint UCL/UCLH Committees on the Ethics of Human Research’.

HEK293 and HEK293T cells were obtained from the American Type Culture Collection and maintained as recommended.

### Preparation of lentiviral constructs

Lentiviruses expression constructs were used to stably transduce CL3^EcoR^ cells. The ORF for each gene of interest was inserted into pLX301 or pLEX-MCS lentiviral vectors by gateway recombinational cloning or by DNA manipulation. Vectors pLX301 (catalogue number 25895) and pLEX-MCS (catalogue number OHS4735) were from Addgene and Thermo Scientific Open Biosystems respectively. Hygromycin resistant expression constructs for LIN9, LIN37, LIN52-S28A and LIN54 were provided by LL and JAD. Since CL3^EcoR^ cells were hygromycin resistant, each of the inserts was sub-cloned into the puromycin resistant pLEX-MCS vector. Full length MYBL2 clone (HsCD00045539) and LIN9 (FL-LIN9) ORF, corresponding to a predicted LIN9 ORF 16 amino acids longer at the amino terminus than the original LIN9 were obtained from DNASU, a plasmid repository (https://dnasu.org/DNASU/). Gateway recombination cloning was used to insert these ORFs into the pLX301 destination vector. Both the constitutively active and the wild type form of FOXM1 were provided by Prof. Rene Medema (Netherlands Cancer Institute, Amsterdam, Netherlands) and sub-cloned into pLEX-MCS lentiviral vector. WT-LIN52-S28 and LIN52-S28E mutants were designed and ordered from GeneArt Gene Synthesis and Services [ThermoFisher Scientific (https://www.thermofisher.com/)]. The ORFs were cloned into pLEX-MCS vector.

### Lentiviral packaging and infection

Lentiviruses were prepared by transfecting 1.5 µg of recombinant lentiviral vector constructs in pLEX-MCS, pLX301 or pGIPZshRNAmir, 1 µg of Gag/Pol expression vector and 1 µg of VSV-G envelope expression vector (pMDG2) into HEK293T cells by FuGENE 6 Transfection reagent (Promega), according to the manufacturer’s instructions.

10^5^ CL3^EcoR^ cells seeded in T-25 flasks were doubly infected with lentiviral supernatants for 24 h at 33.5 ± 0.5 °C to maximise the transduction efficiency. Stably transduced cells were selected using puromycin (2 μg/ml). For the senescence bypass assay, 3.5–5 × 10^4^ stably transduced cells plated in T-75 flasks were cultured at the non-permissive temperature (39 ± 0.5 °C) for 3 weeks. To measure the extent of senescence bypass, the numbers of dense growing colonies were identified by methylene blue staining.

### Gene set analysis

Microarray data on differential gene expression following senescence-induced growth arrest and its bypass were published previously^7^. They are available from Gene Expression Omnibus database accession number GSE24810. The 343 direct p53 target genes were taken from Supplementary Table S3 published in^18^. The DREAM, MMB-FOXM1, and RB-E2F target gene sets were taken from Supplementary Tables S7, S8, and S9, respectively, published in^19^. Venn diagrams were generated using BioVenn^17^. 549 up and 685 downregulated genes, whose expression was reversed upon senescence bypass, correspond to the 816 up and 961 downregulated microarray probes previously published in^7^.

### Immunoblot analysis

CL3^EcoR^ and HMF3S cells were plated at 34 °C and cultured. The following day, the cultures were shifted to 39 °C. 96 h after the temperature shift, each 10 cm dish of cells was lysed in 400 μl ice-cold ELB-SDS buffer (250 mM NaCl, 50 mM HEPES, 5 mM EDTA, 5 mM DTT, 0.1% Triton X-100, 0.1% SDS) supplemented with complete protease inhibitor cocktail (Roche) and phosphatase inhibitors (10 mM NaF, 10 mM β-glycerophosphate and 1 mM Na_3_VO_4_). One parallel dish of cells for each condition was trypsinized and counted to determine the cell number. Lysates corresponding to equal numbers of cells (4.375 × 10^4^ cells per condition) were fractionated on SDS-PAGE gels and transferred to a 0.2 µm nitrocellulose membrane. Membranes were incubated with primary antibodies overnight at 4 °C, washed with TBST and probed with HRP secondary antibodies. The imaging of protein bands was undertaken using ChemiDoc MP imaging system and X-ray films.

### Antibodies

**Table:** 

Target	Resource	Identifier
FOXM1	Cell Signaling Technology	Cat# 5436, RRID: AB_10692483
B-MYB	Millipore	Cat# MABE886
LIN9	Bethyl	Cat# A300-BL2981
LIN37	Santa Cruz Biotechnology	Cat# sc-515686
LIN52	Bethyl	Cat# A300-BL1372
LIN54	Bethyl	Cat# A303-799A, RRID: AB_11218173
RB1	Cell Signaling Technology	Cat# 9309, RRID: AB_823629
pRB^S780^	Abcam	Cat# ab32513, RRID: AB_777635
p107	Santa Cruz Biotechnology	Cat# sc-317, RRID: AB_632093
p130	Santa Cruz Biotechnology	Cat# sc-318, RRID: AB_2175428

## Supplementary Information

Supplementary Figures.

Supplementary Table 1.
