# Supplementary material for: Simultaneous expression of MMB-FOXM1 complex components enables efficient bypass of senescence
Source: Sci Rep. 2021 Nov 2;11:21506. doi: 10.1038/s41598-021-01012-z (PMC8563780; doi:10.1038/s41598-021-01012-z)

FOXM1

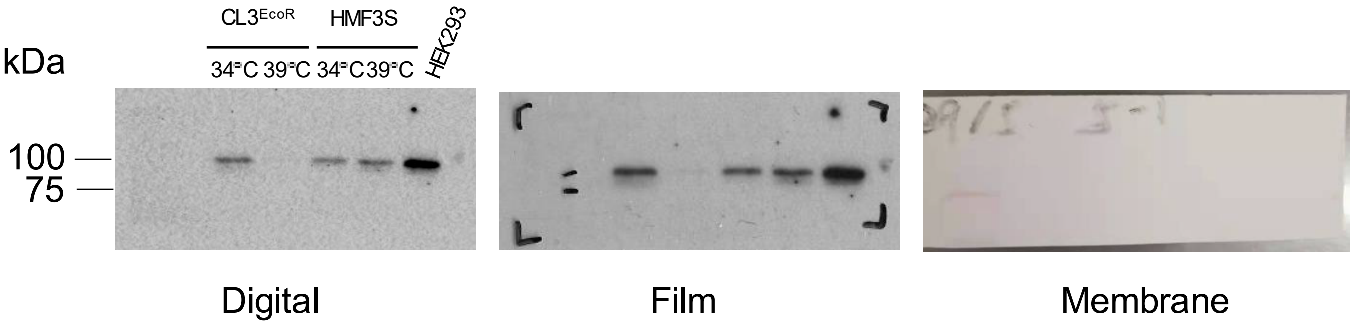
CL3EcoR
HMF3S
HEK293
kDa
34°C 39°C 34°C 39°C
100
75
Digital
Film
Membrane

B-MYB

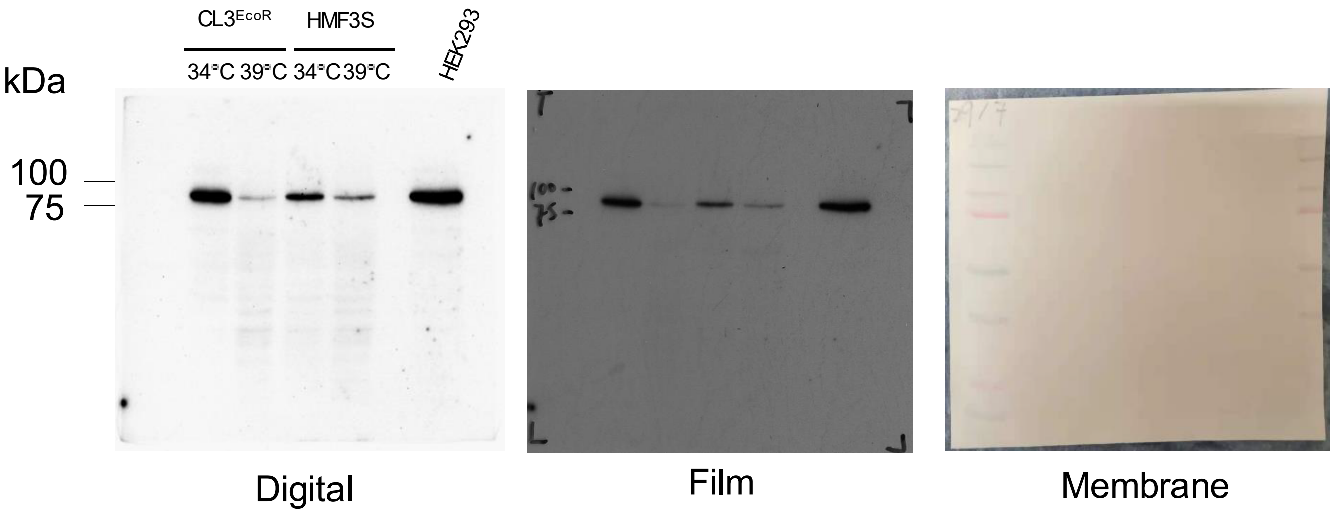
CL3EcoR
HMF3S
34°C 39°C 34°C 39°C
HEK293
kDa
100
75
Digital
Film
Membrane

LIN9

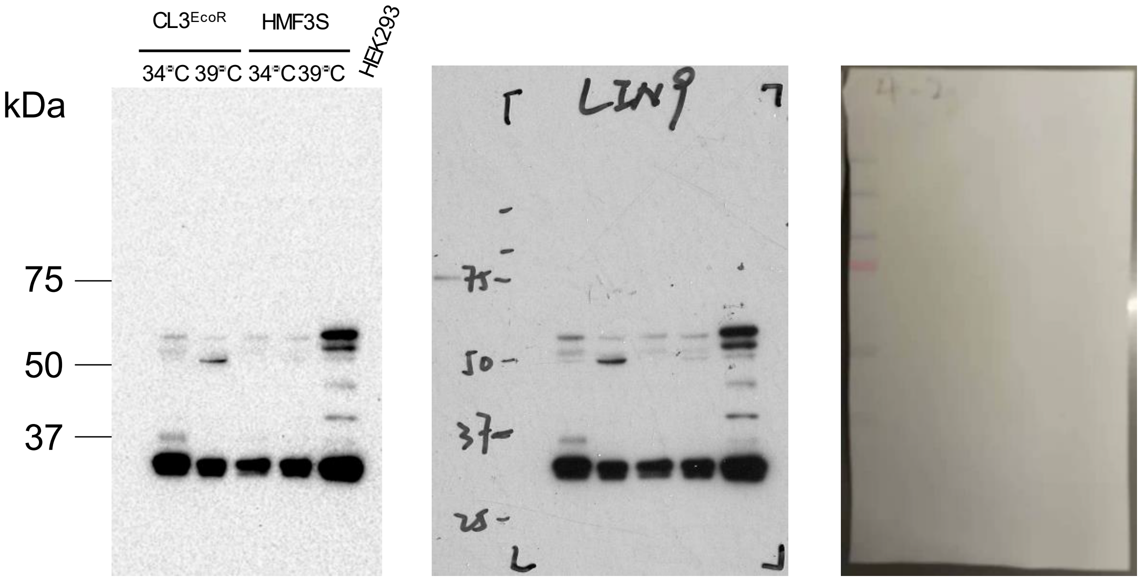

Digital Film Membrane

LIN37

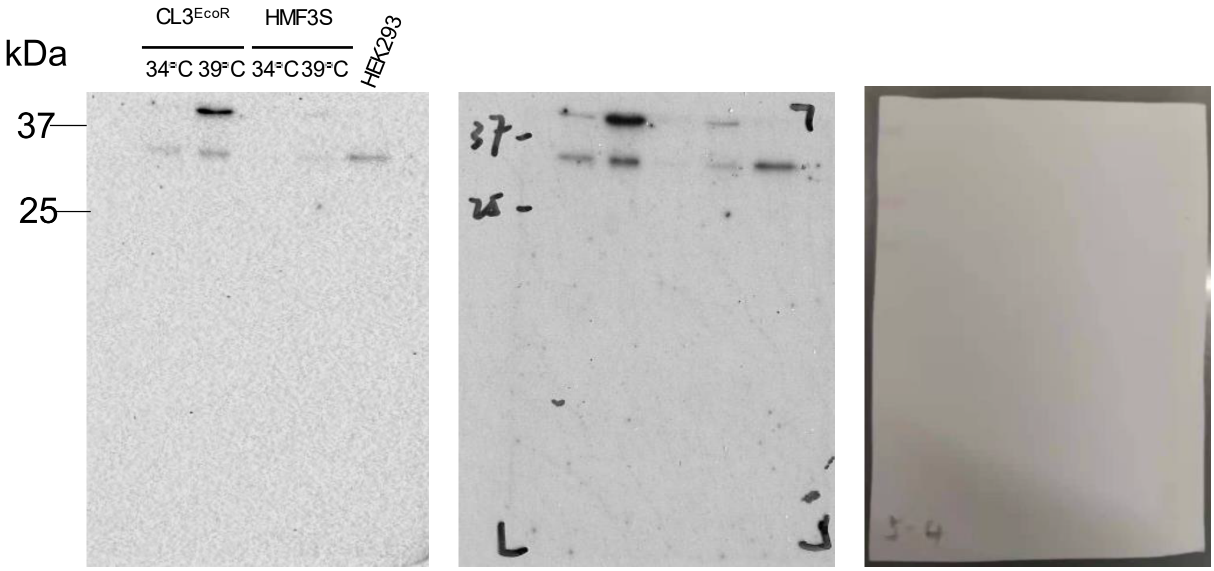

Digital Film Membrane

LIN52

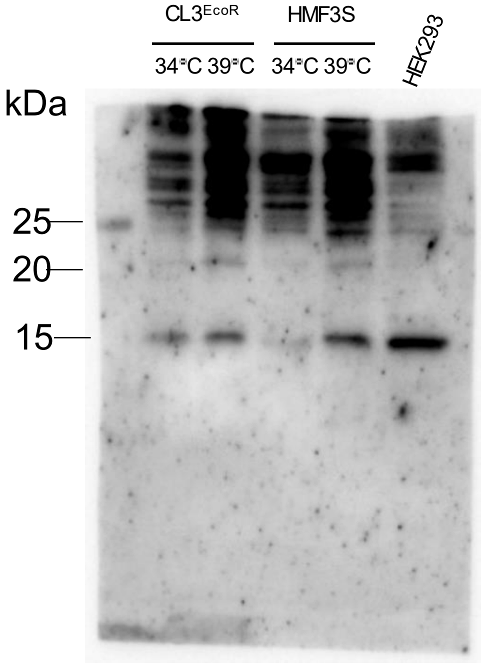

Digital

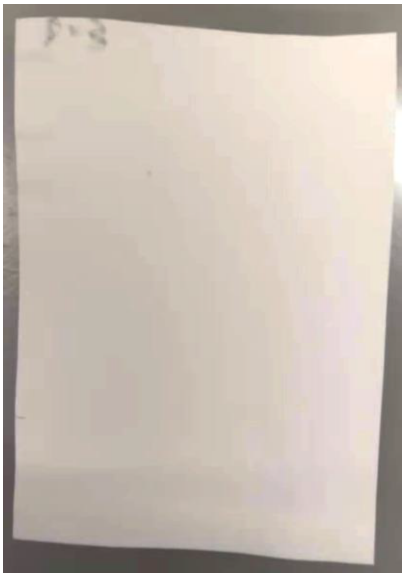

Membrane

LIN54

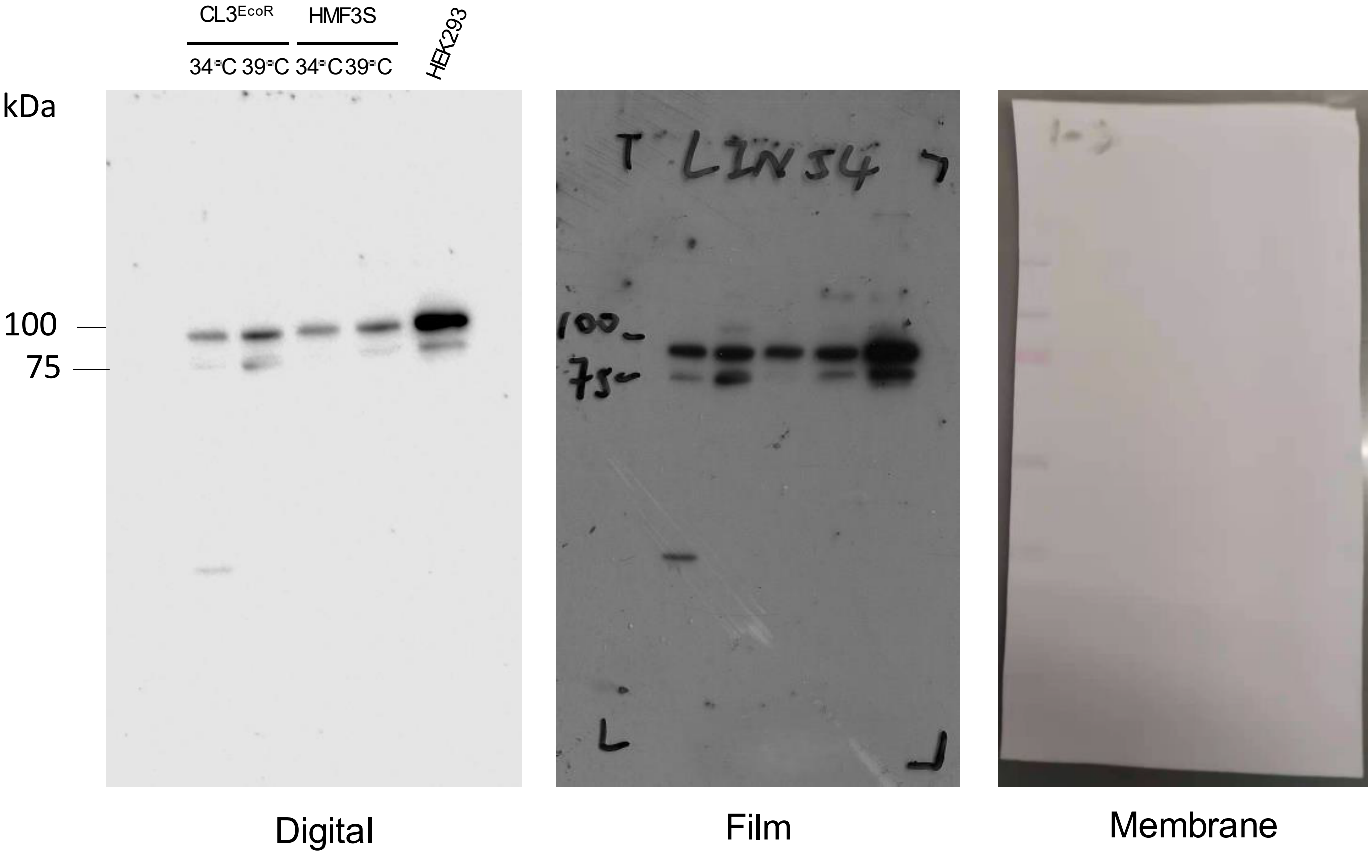
CL3EcoR
HMF3S
34°C 39°C 34°C 39°C
HEK293
kDa
100
75
Digital
Film
Membrane

DYRK1A

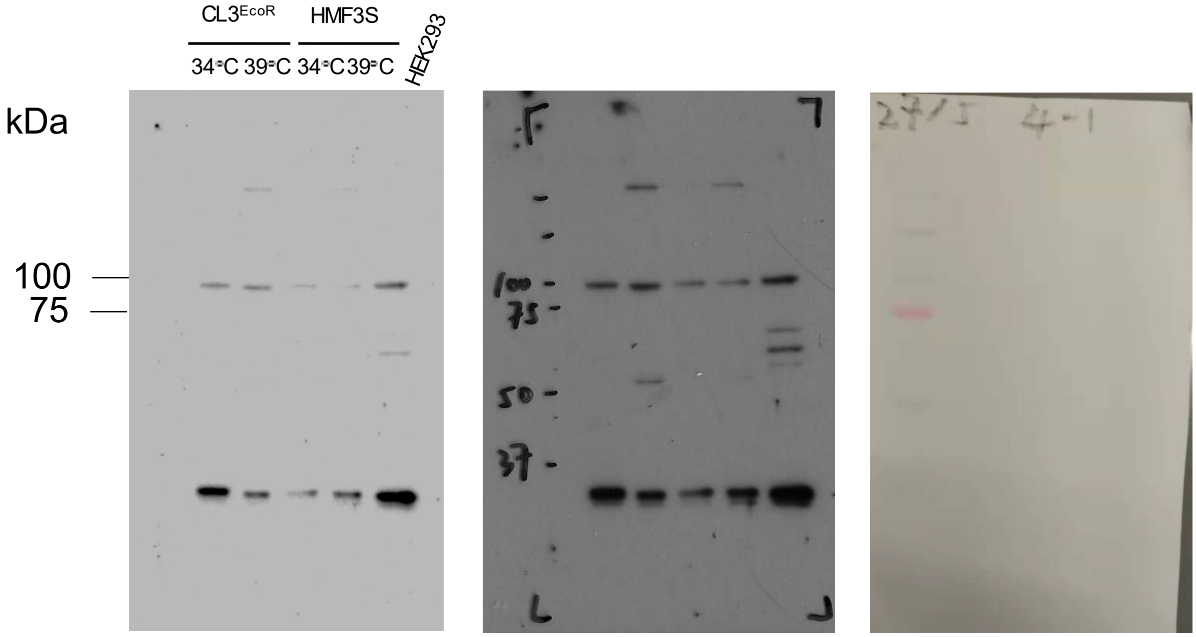

Digital Film Membrane

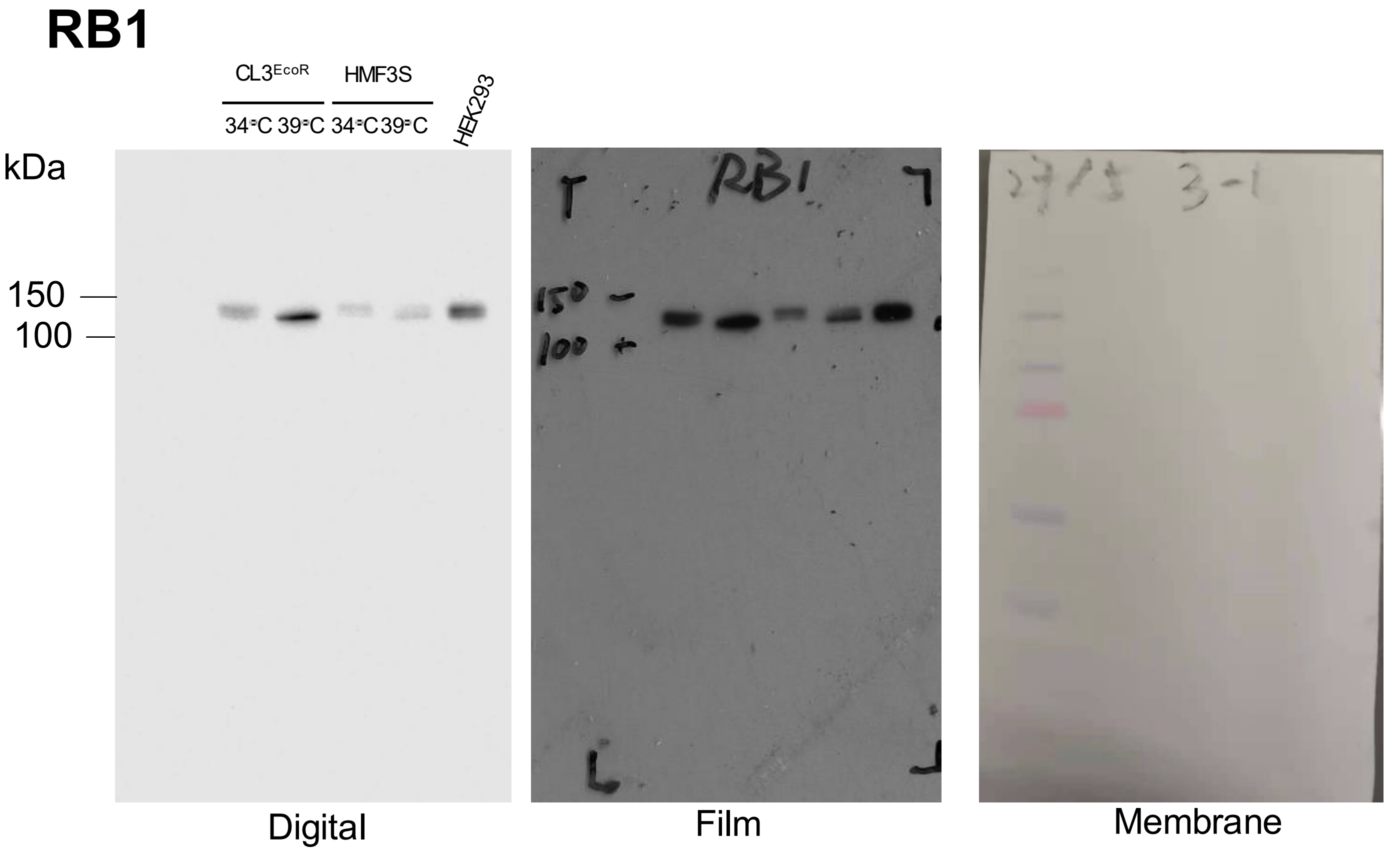
RB1
CL3EcoR
HMF3S
34°C 39°C 34°C 39°C
HEK293
kDa
150
100
Digital
Film
Membrane

pRB^{S780}

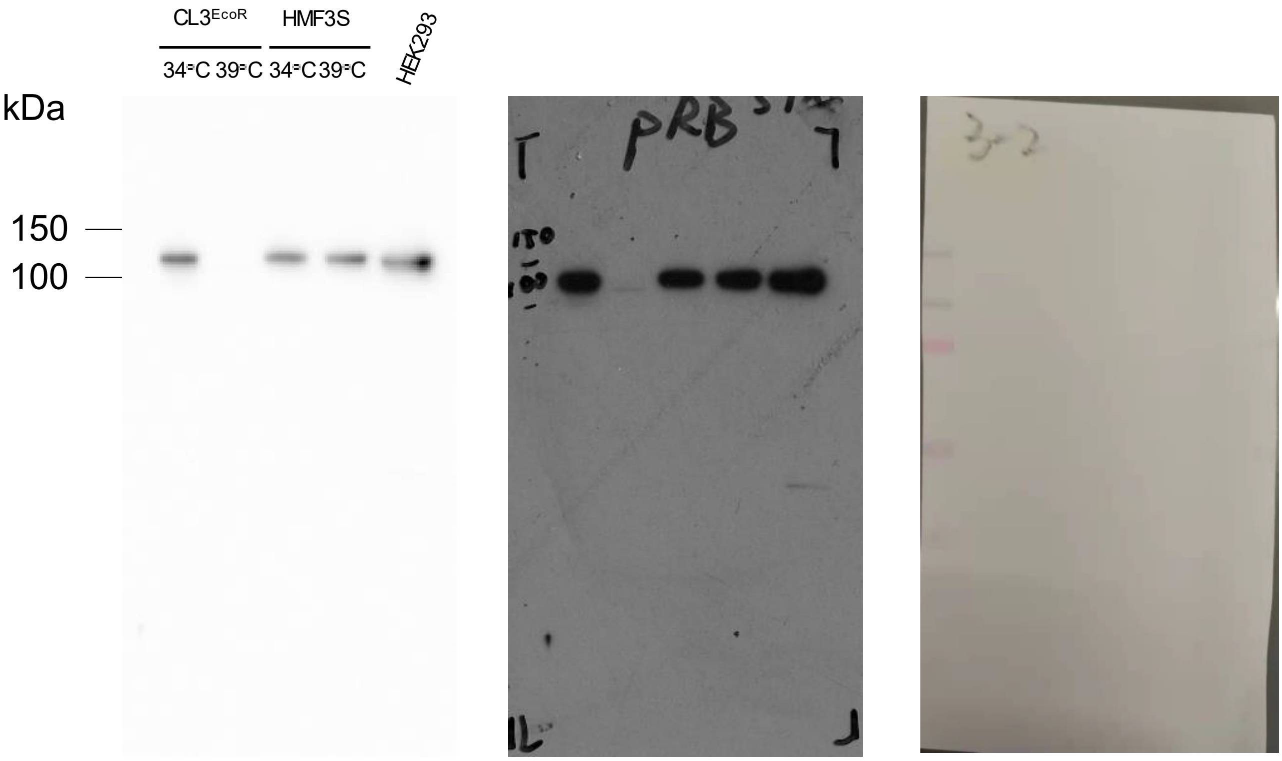

Digital Film Membrane

p107

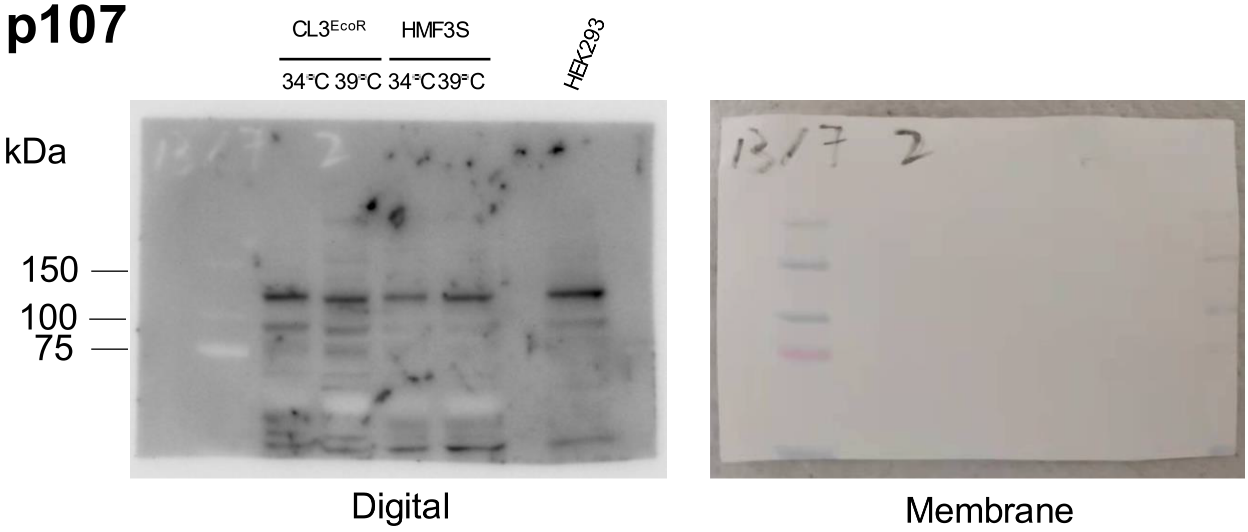
CL3^EcoR
HMF3S
34°C 39°C 34°C 39°C
HEK293
kDa
150
100
75
Digital
Membrane

p130

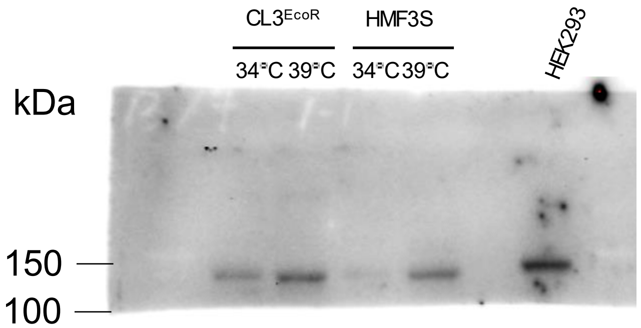

Digital

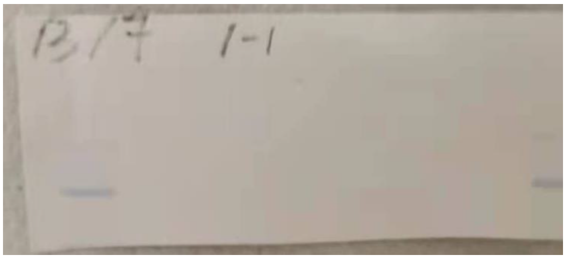

Membrane

Ponceau staining

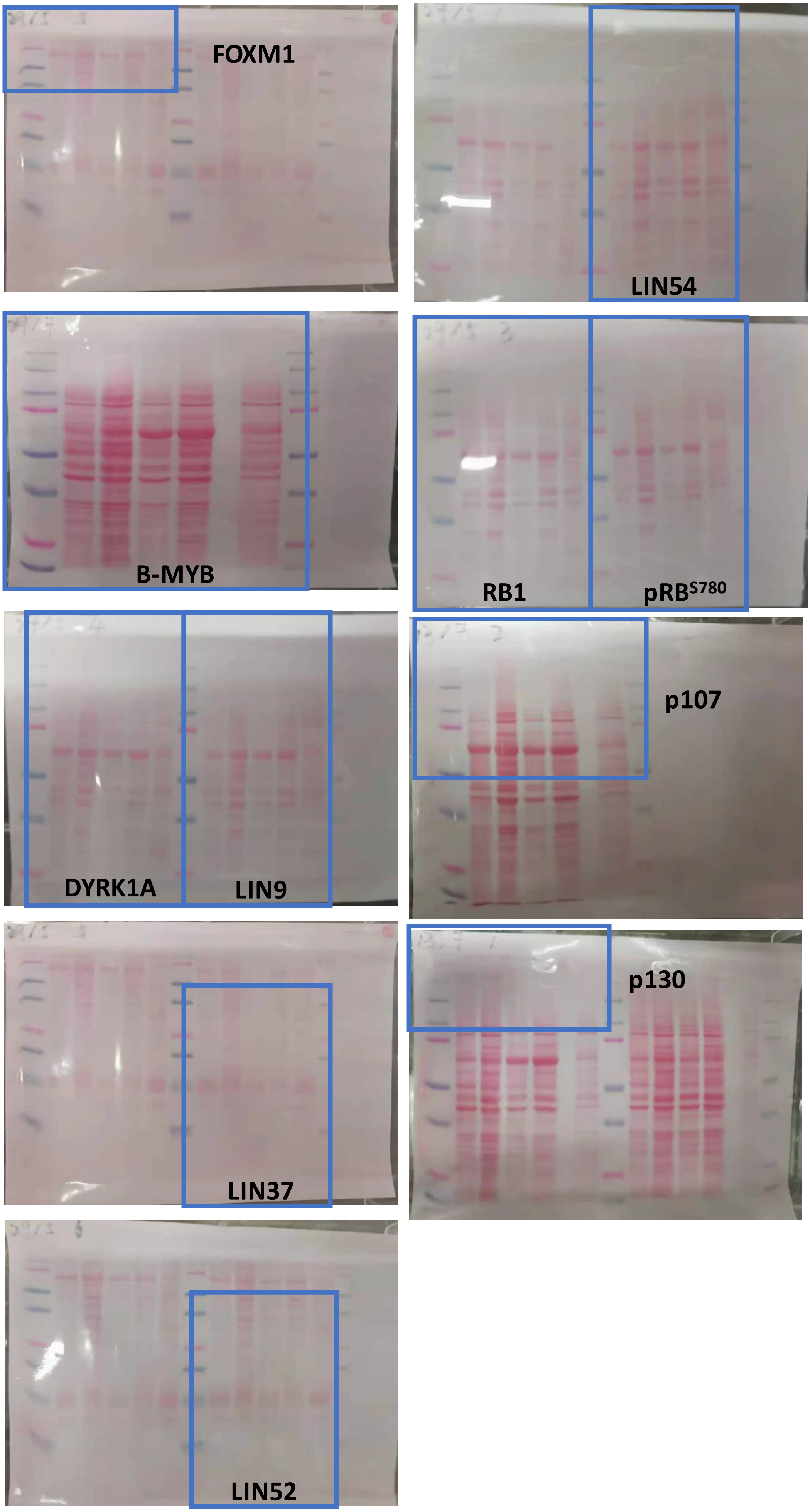

Supplement: Supplementary file 1 — Supplementary Figures. [file 41598_2021_1012_MOESM1_ESM.pdf]
